# It takes two to tango: Preserving daptomycin efficacy against daptomycin-resistant MRSA using daptomycin-phage co-therapy

**DOI:** 10.1128/spectrum.00679-24

**Published:** 2024-10-29

**Authors:** Casey L. Madison, Anja S. J. Steinert, Corrin E. Luedeke, Neeka Hajjafar, Prakhar Srivastava, Andrew D. Berti, Arnold S. Bayer, Razieh Kebriaei

**Affiliations:** 1 P3 Research Laboratory, Division of Pharmaceutics and Pharmacology, College of Pharmacy, The Ohio State University, Columbus, Ohio, USA; 2 Department of Pharmacy Practice, College of Pharmacy, Wayne State University, Detroit, Michigan, USA; 3 David Geffen School of Medicine, University of California Los Angeles, Los Angeles, California, USA; 4 The Lundquist Institute at Harbor-UCLA Medical Center, Torrance, California, USA; Innovations Therapeutiques et Resistances (INTHERES), Université de Toulouse, Toulouse, France

**Keywords:** antibiotic resistance, bacteriophage therapy, *Staphylococcus aureus*

## Abstract

Daptomycin (DAP) is a cyclic lipopeptide antibiotic that targets the cell membrane and is commonly used for the treatment of multidrug-resistant *Staphylococcus aureus* infections, especially methicillin-resistant strains (MRSA). Emergence of DAP resistance during DAP therapy is increasingly reported; however, the mechanisms by which resistance occurs are diverse. Using phages in combination with antibiotics is of high interest to circumvent antibiotic resistance, due to their minimal side effects, accessibility, and synergistic effects with antibiotics. Here, we have investigated the relationship between DAP resistance and sensitivity to phage Sb-1 in a cohort of 14 clinical MRSA strains. This cohort consists of four isogenic pairs (a clinical DAP-susceptible parent and its isogenic DAP-resistant variant isolated during DAP treatment), and three DAP-susceptible/DAP-resistant strain pairs are created by traditional serial passaging in the presence of increasing DAP concentrations. Efficiency of plating (EOP) and bacteriophage plaque sizes were recorded, and synergy between the antibacterial agents was tested using the phage-antibiotic combination (PAC) checkerboard method. Plaque sizes and EOP significantly increased (*P* = 0.019 and *P* = 0.008, respectively) as strains developed resistance to DAP. The PAC checkerboards conveyed less antagonistic patterns in DAP-resistant mutant strains compared with their DAP-susceptible counterparts. Antagonism was displayed in 71.4% (5/7) of the DAP-susceptible strains, while it was only present in 14.3% (1/7) of the DAP-resistant mutants. Transmission electron microscopy images showed significantly thicker cell walls in DAP-susceptible strains exposed to DAP monotherapy compared with either growth control or DAP-phage (*P* = 0.0002 and 0.0007, respectively). These data indicate increased sensitivity to phage Sb-1 concurrent with the emergence of DAP resistance.

**IMPORTANCE:**

Multidrug-resistant *Staphylococcus aureus* is a threat to the health care system, especially cross-resistance between daptomycin (DAP) and glycopeptides through various mutations such as *mprF* (which is involved in the modification of membrane phospholipids in some bacteria) and yycG (part of a two-component regulatory system in bacteria that is important for regulating cell wall biosynthesis and other cellular processes) has been reported previously. Our current study shows adjunctive treatment with phage in DAP-resistant strains will lead to synergistic activity and larger phage plaque sizes, translating to elevated lytic performance. The addition of bacteriophage to standard-of-care antibiotic therapies for multidrug-resistant *S. aureus* infections has the potential to hinder, and possibly revert, resistance to antibiotics. Applying this strategy can potentially lead to the preservation of the current antibiotics. Verification of this salutary outcome in relevant *ex vivo* and *in vivo* models of endovascular infections is required to validate translatability.

## INTRODUCTION

*Staphylococcus aureus* is an organism that causes a broad range of clinical pathologies ranging from skin and soft-tissue infections to life-threatening bloodstream infections in both community and nosocomial settings (1, 2). Bacteriophages (“phages”) are viruses that infect bacteria and are specific to individual bacterial species. Because of their narrow host range, they can target pathogenic bacteria, while the normal microbial flora is only minimally disrupted, if at all (3, 4). Optimal phages for therapeutic use are those that are lytic, all of which are bactericidal (4). Targeted phage therapy with a lytic phage is therefore a highly promising avenue for addressing the increasing problem of antimicrobial resistance. Here, we have used a lytic staphylococcal phage that covers a broad host range of *S. aureus,* including methicillin-resistant strains (MRSA) and has been used clinically since 1977 (1, 5).

Daptomycin (DAP) is a cyclic lipopeptide that targets the cell membrane and is commonly used for the treatment of recalcitrant MRSA infections. This antibiotic was particularly interesting to us because its mechanism of action closely resembles that of some host defense peptides. Key similarities include membrane disruption, cationic properties, membrane targeting, and the induction of membrane stress. Therefore, exposure to endogenous host defense peptides may significantly influence the selection of strains with intrinsically higher DAP MIC (Minimum Inhibitory Concentration) values (6, 7).

Of note, emergence of DAP resistance during DAP therapy has led to DAP treatment failures and increased mortality (8, 9). While the Clinical Laboratory Standards Institute defines a minimal inhibitory concentration of ≤1 µg/mL as DAP-susceptible, no specific value has been defined for DAP resistance (10). Isolates with MIC >1 µg/mL are technically defined as “DAP-nonsusceptible.” The terms “resistant” and “nonsusceptible” are used interchangeably to denote the same concept. We will be referring to DAP-resistant strains and DAP-susceptible strains as DAP-R and DAP-S, respectively.

Antibiotic-phage combinations have been researched as an alternative therapy to target multidrug-resistant bacteria (1, 11). In this investigation, we have compared phage sensitivity and DAP-phage combination synergy among a cohort of well-characterized isogenic pairs of DAP-S *S. aureus* strains and their DAP-R mutants.

## RESULTS

**FIG 1:**
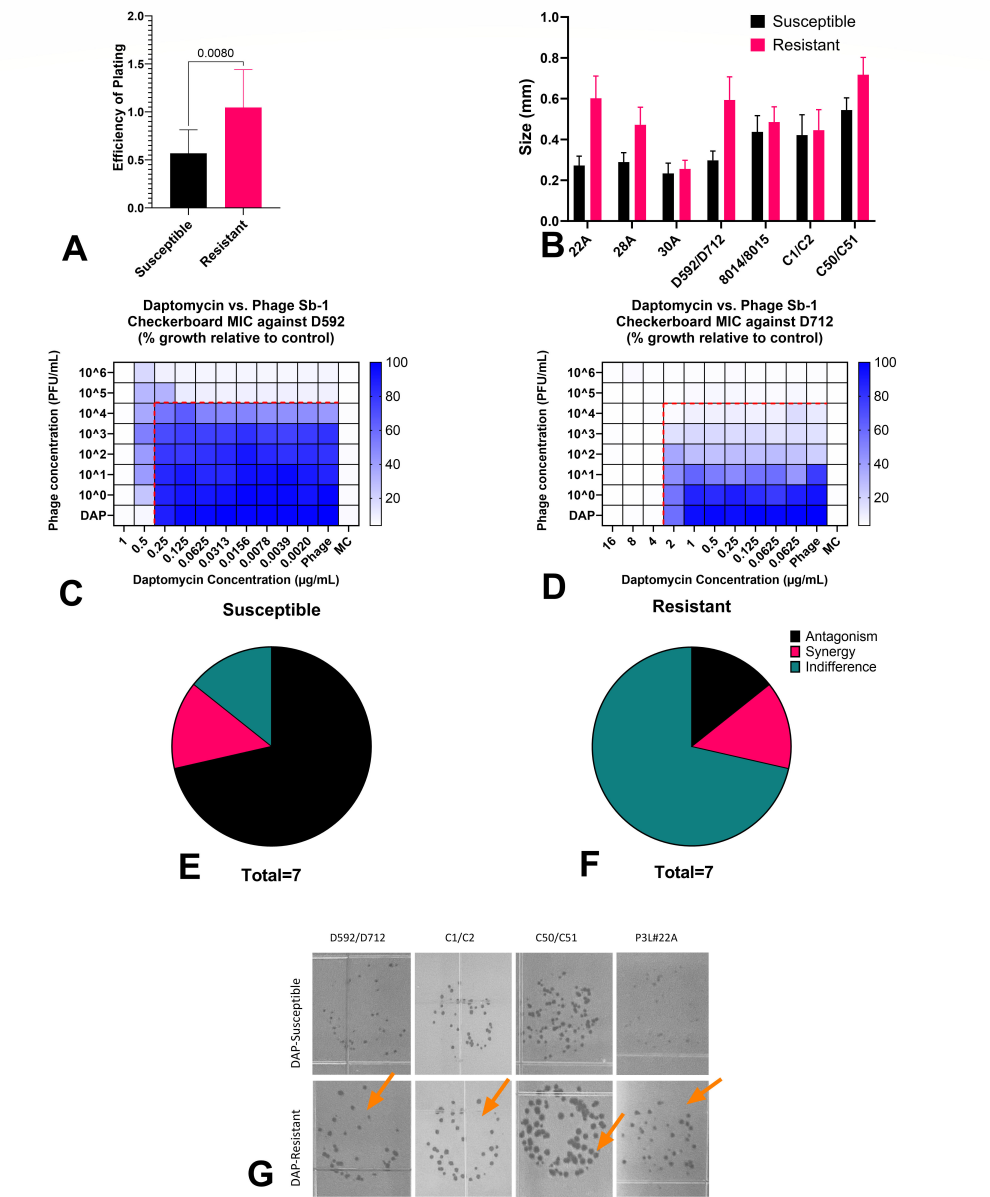
.Comparison of phage sensitivity between daptomycin-susceptible (DAP-S) and daptomycin-resistant (DAP-R) strains. (**A**) Mean difference in the efficiency of plating. (**B**) Phage Sb-1 plaque sizes. (**C**) Depiction of synergistic patterns (phage-daptomycin combination) in DAP-S compared with (**D**) DAP-R strain. (**E, F**) Summary of phage-antibiotic checkerboard results with daptomycin and phage combination. (**G**) The plaque size change (orange arrows) in DAP-R vs. DAP-S.

To assess the difference in phage sensitivity, we analyzed plaque size and efficiency of plating (EOP) differences between resistant and parent strains. Additionally, we evaluated antagonistic pattern changes in DAP-R strains relative to their parent. Significantly higher EOP was observed with DAP-R strains (*P* = 0.008) (Fig. 1A). Moreover, significantly increased plaque sizes were also found with DAP-R strains compared with the respective parent strains (*P* = 0.019) (Fig. 1B and G).

The phage-antibiotic checkerboard assays (Fig. 1C and D) depicted a greater synergistic pattern in the DAP-R variants compared with the respective isogenic parent. Also, more antagonism cases were observed in DAP-S vs. DAP-R strain pairs. While synergy was only demonstrated in one strain pair (P3L22A, both susceptible and resistant), there was less antagonism in DAP-resistant isolates. Thus, 71.4% (5/7) of the DAP-susceptible isolates had antagonistic checkerboard patterns, while such patterns were only present in 14.3% (1/7) of the DAP-R mutants (Fig. 1E and F). Out of the five parental DAP-S strains that displayed antagonism against the combination, four of their DAP-resistant mutants demonstrated “indifference” (defined as growth exactly at MIC values). The frequency of resistance to phages in DAP-S and DAP-R strains was generally in the range of 10^−3^–10^−5^ for parent and mutant strains, respectively.

Table 1 lists the genetic changes in DAP-R strains relative to their DAP-S parents. As anticipated, *mprF* mutations are common among DAP-R isolates, consistent with their role in DAP-R and their low barrier to genetic drift (12, 13). Additional mutations associated with the transition to a DAP-R phenotype were identified in *walK, rpoC,* and *cls2* (7).

**TABLE 1:** Summary of genetic information and GenBank accession numbers

Strain	Description	Accession	Reference or source
CUBIST-1	ST5-MRSA-II index clinical isolate	CP155817	(14)
⤷ CUBIST-2	⤷ Sequential clinical isolate, *mprF*^L826F^	CP168156	
CUBIST-17	ST5-MRSA-II index clinical isolate	CP155815	(14)
⤷ CUBIST-18	⤷ Sequential clinical isolate, *mprF*^L341S^ *cls2*^R295C^	CP155816	
CUBIST-50	ST225-MRSA-II index clinical isolate	CP156175	(14)
⤷ CUBIST-51	⤷ Sequential clinical isolate, *mprF*^T345I^	CP168095	
D592	2010 ST5-MRSA-II index clinical isolate	CP040665	(15)
⤷ D712	⤷ Sequential clinical isolate, *mprF*^L341S^	CP168754	
R8014	2012 ST5-MRSA-II index clinical isolate	CP156697	(16)
⤷ R8015	⤷ Sequential clinical isolate, *mprF*^L826F^*walK*^Y440D^	CP156750	
P3L22	ST30-MSSA clinical isolate	CP168224	This study
⤷ P3L22A	⤷ *In vitro*-derived mutant, *rpoC*^Y62C^	CP168436	
P3L28	ST840-MRSA-IVg clinical isolate	CP168717	This study
⤷ P3L28A	⤷ *In vitro*-derived mutant, *mprF*^A96V^	CP168440	
P3L30	ST544-MSSA clinical isolate	CP168221	This study
⤷ P3L30A	⤷ *In vitro*-derived mutant, *mprF*^P314T^	CP168437	

**FIG 2:**
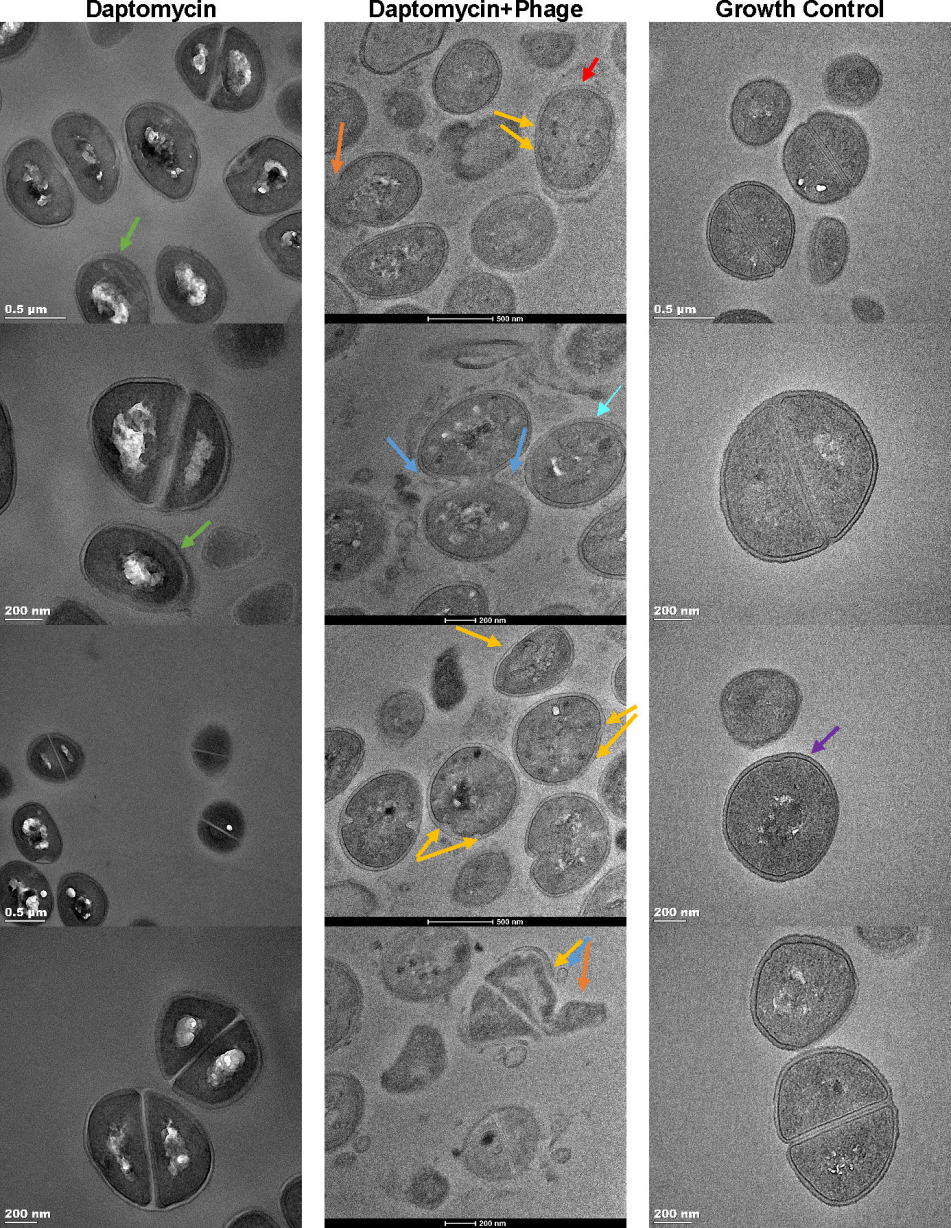
Transmission electron microscopy (TEM) images of three main treatment regimens, daptomycin monotherapy (DAP = 0.5 × MIC), daptomycin + phage (DAP = 0.5 × MIC, phage = 10^6^ PFU/mL), and growth control. Arrows: orange: lysis of intracellular contents. Yellow: irregular perturbations (without division) in the cell wall due to phage lysin in DAP + phage treatment. Green: thicker cell wall when exposed to daptomycin single treatment, Cyan: cell wall thickness after exposure to both phage and daptomycin, Purple: intact cell wall in growth control treatment. Blue: phage particles at the division septum. Red: distorted cell wall.

Transmission electron microscopy (TEM) images showed thicker cell walls (CWs) for D592 (DAP-S) strain exposed to DAP monotherapy vs. either DAP-phage or GC treatments (*P* = 0.0007 and 0.0002, respectively). While there was no significant difference in CW thickness between GC and DAP-phage treatment (Fig. 2; Fig. S1).

## DISCUSSION

This study suggests that as the DAP-susceptible parent strains develop DAP resistance (either clinically during DAP therapy or *in vitro* during serial DAP passage), they become more phage-sensitive, as evidenced by higher phage concentrations observed in plaque assays (17). Larger plaque sizes were also observed in the resistant isolates, which indicates higher phage lytic activity and higher phage sensitivity (18–20). Furthermore, increased synergy between phage Sb-1 and DAP was observed with DAP-resistant strains. This observation aligns with previous research indicating that CW metabolism is upregulated in DAP-resistant isolates compared with their susceptible parents; this includes enzymes involved in wall teichoic acid (WTA) biosynthesis. Of note, WTA has been identified as the main location for the majority of phage receptors in *S. aureus* (7, 21). The thickened CW phenotype in the DAP-resistant strain is likely linked to an upregulation of the tag operon, which correlates with the increased production of WTA.

While DAP is still commonly used for the treatment of difficult MRSA infections, the emergence of DAP-R during DAP therapy has been well documented (22). Resistance to phage can also occur through a variety of mechanisms, such as blockage of phage adsorption through blockage of phage receptors or production of competitive inhibitors (exopolysaccharides) (23). It is probable that fitness to DAP results in the modification of cell surface receptors (structurally) or leads to increase in such receptors. Alternatively, this fitness may cause reduced production of the competitive inhibitors, resulting in better phage binding and propagation (24).

It is interesting that, although all DAP-R strains were less likely to exhibit antagonism with Sb-1 phage than their DAP-S parents, the only DAP-R strain to exhibit outright DAP-phage synergy, P3L22A, was also the only DAP-R strain to develop DAP-R without an *mprF* mutation. MprF mutations resulting in DAP-R increase the activity of the bifunctional enzyme, either increasing the rate of cell membrane phosphatidylglycerol (PG) lysinylation on the intracellular leaflet or increasing the rate of lysyl-phosphatidylglycerol (LPG) flipping to the extracellular leaflet (13). PG in the intracellular leaflet acts as a substrate in the synthesis of WTA, whereas PG in the extracellular leaflet acts as a substrate in the synthesis of lipoteichoic acids (LTA). Teichoic acids serve as both osmoprotectants and as the primary attachment site for bacteriophages (25). PG-to-LTA/WTA turnover is robust and essential for staphylococcal viability. During exponential growth, the entire PG pool turns over twice per generation time at the cost of up to 40% of the cell’s total ATP expenditure (26). It is therefore tempting to speculate that increased LPG content might alter WTA and LTA content and thus reduce the availability of the primary phage receptors in every mutant strain except for P3L22A. However, staphylococci are exquisitely sensitive to teichoic acid content and use this as a signal to modulate both cell membrane fluidity and CW crosslinking (27, 28). The observation that DAP-R strains containing *mprF* mutations still demonstrate less antagonism than their parent stains suggests that the pleiotropic changes to cell membrane and CW physiology associated with the transition to DAP-R more than compensate for the potential receptor availability impact of WTA and LTA content change. The increase in CW thickness observed in the D592 strain (DAP-S) during exposure to DAP monotherapy is significant, whereas this increase is not seen with GC or DAP-phage treatments. This thickening could be a stress response and an indication of the development of a DAP-resistant phenotype due to DAP exposure/pressure, involving CW remodeling that may include enhanced production of peptidoglycan and WTA, leading to an increased number of phage receptors.

The limitations of this study include (i) there is a finite number of established DAP-S parental isolates and their respective DAP-R strain pairs. Furthermore, only a fraction of those pairs are likely to be sensitive to bacteriophage Sb-1. For that reason, the sample size listed in this study is small, and larger cohorts are needed for future studies. (ii) There is a need for an *in vivo* investigation of our results and evaluation of exogenous phage exposures in the presence of antimicrobial peptides. It will also be valuable to test the potency of DAP and phage combinations over a more extended interaction period (29).

Overall, our data indicate that the development of resistance to antibiotic DAP is accompanied by collateral sensitivity to phage. We have demonstrated that DAP-phage combinations exhibit synergistic interactions *in vitro*. This phage-DAP combination has the potential to salvage patients with recalcitrant DAP-R infections. Of course, these *in vitro* results require verification in relevant *in vivo* models.

## MATERIALS AND METHODS

### Bacterial and phage strains

Seven isogenic DAP-S and DAP-R *S. aureus* (MRSA) isolate pairs were used. Four pairs are clinical patient isolates (D712/D592, 8014/8015, C1/C2, C50/C51). For the other three pairs, DAP-R mutants were created through serial passaging from their parent strains (P3L22A, P3L28A, and P3L30A). The bacteriophage used was Sb-1 phage purchased from Eliava Phage Institute, Tblisi, Georgia. Sb-1 is a myophage categorized as Herelleviridae, GenBank accession no HQ163896.

### Experimental methods

All experiments used propagated phage Sb-1 (second-generation propagation). The three strains created through serial passaging were created similarly to previously described methods (30). The modified plaque assay was conducted to calculate EOP and measure bacteriophage plaque sizes (31, 32). Phage plaque sizes were measured using ImageJ software (v.1.8.0) and recorded as an average of at least 10 independent repeats (33). TEM was performed to visualize the morphology of bacterial strains with or without exposure to phage as reported previously (34). Additional details are available in supplementary material.

## SUPPLEMENTAL MATERIAL

Supplemental material spectrum.00679-24-s0001.docx Materials and methods; Fig. S1.

ASM does not own the copyrights to Supplemental Material that may be linked to, or accessed through, an article. The authors have granted ASM a non-exclusive, world-wide license to publish the Supplemental Material files. Please contact the corresponding author directly for reuse.
