# Supplementary material for: It takes two to tango: Preserving daptomycin efficacy against daptomycin-resistant MRSA using daptomycin-phage co-therapy
Source: Microbiol Spectr. 2024 Oct 29;12(12):e00679-24. doi: 10.1128/spectrum.00679-24 (PMC11619598; doi:10.1128/spectrum.00679-24)
Supplement: Supplemental material — Materials and methods; Fig. S1. [file spectrum.00679-24-s0001.docx]

**Supplementary Material:**

*Antibacterials and Media*

Daptomycin (DAP) (SAGENT Pharmaceuticals, Schaumburg, IL, USA) was purchased commercially. Tryptic Soy Agar (TSA) (BD, Sparks, MD, USA) plates were used to grow all strains for MIC testing. Mueller-Hinton broth II (MHB) (BD, Sparks, MD, USA) supplemented with an additional 25mg/L of calcium (CaCl_2_, total of 50mg/mL) was used for all MIC and checkerboard tests. Heart Infusion Broth (HIB) (BD, Sparks, MD, USA) was used for liquid culture growth of *S. aureus* for phage related tests. Phage plating used Heart Infusion Broth (HIB) (BD, Sparks, MD, USA) with added agar (Oxoid, Hampshire, UK) at 1.5% or 0.7% for petri plates and overlay agar respectively.

*Modified plaque assay*

100μL of an overnight culture of bacteria (~16 hours) was combined with 0.7% molten agar overlay and poured onto a 1.5% agar HIB plate. Once dry, three drops of serially diluted phage were added to the plate, and plates were incubated at 36±1°C overnight. EOP was derived from the number of formed plaques with the test strain relative to the host strain.

*Checkerboard analysis*

Phage-antibiotic combination (PAC) checkerboard analyses were performed to determine the potency of phage Sb-1 and DAP combinations in comparison to their individual minimum inhibitory concentrations (MIC). MIC values of DAP for all isolates were determined in duplicate by broth microdilution. The absorbance values were used to create a heat map (Epoch 2 Microplate Reader, BioTek, USA) showing the percentage of the bacterial population relative to the growth control standard and blank tests.

*Whole genome sequencing*

Genome sequence was determined by Illumina HiSeq 150×2 paired end technologies at the Infectious Diseases Institute Genomics and Microbiology Solutions Center at The Ohio State University, assembled using SPADES and annotated by the NCBI Prokaryotic Genome Annotation Pipeline (PGAP). Sequences have been deposited to GenBank under BioProject number PRJNA1109366.

*Transmission electron microscopy*

Bacterial pellets (10^8^ CFU/mL) were fixed in 2.5 % glutaraldehyde in 0.1 M sodium cacodylate buffer pH 7.0 at 4 ^o^C. Glutaraldehyde was removed and pellets were washed three times 10 minutes each with sodium cacodylate buffer pH 7.0.  Pellets were then stained with 1 % Osmium Tetroxide in 0.1 M sodium cacodylate buffer for 2 hours at 4 ^o^C.   Pellets were stained with 0.5 % uranyl acetate in water at 4 ^o^C overnight.   The pellets were incubated in 100 % resin for 2 hours at room temperature.  After two hours, the samples were placed in a 60 ^o^C oven for 10 minutes and another 100 % resin exchange was performed. Resin blocks were sectioned into 70 nm sections, sections were placed on 300 mesh copper grids, and then imaged on a Tecnai F20 TEM operated at an accelerating voltage of 200 kV.


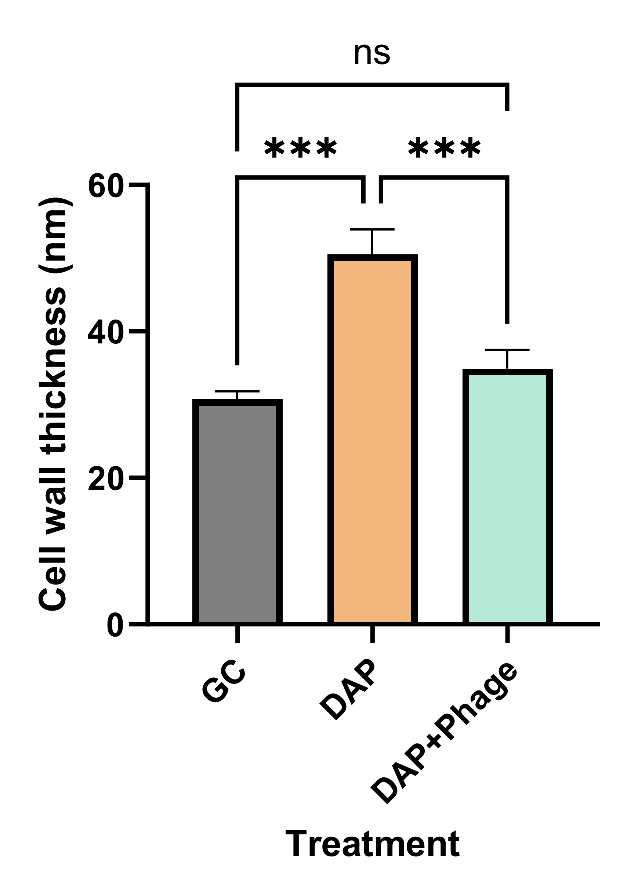


**Figure S1.** Significant differences in cell wall thickness (based on TEM images) between GC (growth control), DAP monotherapy and DAP-Phage treatments.
